# Transcriptome Analysis of Monocytes and Fibroblasts Provides Insights Into the Molecular Features of Periodontal Ehlers-Danlos Syndrome

**DOI:** 10.3389/fgene.2022.834928

**Published:** 2022-04-28

**Authors:** Zhuoyi Liao, Tian Zhao, Ningxiang Wang, Jiaqi Chen, Weibin Sun, Juan Wu

**Affiliations:** ^1^ Department of Periodontology, Nanjing Stomatological Hospital, Medical School of Nanjing University, Nanjing, China; ^2^ Central Laboratory of Stomatology, Nanjing Stomatological Hospital, Medical School of Nanjing University, Nanjing, China; ^3^ Department of Stomatology, Nanjing Hospital of Chinese Medicine, Nanjing University of Traditional Chinese Medicine, Nanjing, China

**Keywords:** Ehlers-Danlos syndrome, periodontitis, transcriptomics, monocytes, RNA-sequencing

## Abstract

Periodontal Ehlers–Danlos syndrome (pEDS) is a rare hereditary disorder characterized by severe early-onset periodontitis with premature tooth loss, pretibial hyperpigmentation, and skin fragility. It is caused by mutant variants in the *C1R* and *C1S* genes that result in C4 cleavage and local complement cascade activation, as well as other possible consequences. However, the exact functional consequences of this activation remain unclear. To shed light on molecular mechanisms underlying pEDS and to identify novel molecular targets that may expand treatment strategies, we performed transcriptome profiling by RNA sequencing of monocytes and gingival fibroblasts from two patients with pEDS. Compared to normal controls, differential expression of genes was found only in monocytes but not gingival fibroblasts. Most of the significant genes were enriched in biological processes such as neutrophil-mediated immunity, response to bacterium, TNF-α and IL-17 pathway which are related to inflammation response and immune response. In disease ontology enrichment analysis, genes related to periodontal host defense, inflammatory response, skin disease, and vascular development, including *MMP9*, *VEGFA*, *IL10*, *IL1A*, *IL1B*, *IL2RA,* and *IL6*, were significantly enriched and also validated by qPCR and ELISA. Overall, the present study provides the transcriptomic data of pEDS for the first time and the distinct molecular features in monocytes of pEDS might serve as a tool to better understand the disease.

## Introduction

The Ehlers–Danlos syndromes (EDS) are a clinically and genetically heterogeneous group of heritable connective tissue disorders characterized by joint hypermobility, skin hyperextensibility, and tissue fragility (Malfait et al., 2017). In 2017, The International EDS Consortium proposed a revised EDS classification (Malfait et al., 2017), and currently, 14 subtypes of EDS are recognized (Blackburn et al., 2018). Among all subtypes of EDS, periodontal Ehlers–Danlos syndrome (pEDS) (also known as EDS type VIII, OMIM#130080) is a specific EDS subtype caused by autosomal dominant pathogenic variants in complement 1 subunit genes *C1R* and *C1S*, with early severe periodontitis as the predominant clinical feature (Kapferer-Seebacher et al., 2016). The other clinical manifestations of pEDS include lack of attached gingiva, pretibial hyperpigmentation, skin fragility with abnormal scars, and easy bruising (Malfait et al., 2017). Treatment of pEDS remains a huge challenge. Most of the current studies published have focused on describing the clinical features of the pEDS and/or identifying genetic variants (Wu et al., 2018; Kapferer-Seebacher et al., 2021). The pathogenesis of pEDS is only partly understood. From previous research (Kapferer-Seebacher et al., 2016), it is confirmed that pEDS is caused by pathogenic variants *C1R* (type 1, MIM 613785) and *C1S* (type 2, MIM 120580) genes, which encode the C1r and C1s subunits of the first step of the classical complement cascade, a major antimicrobial pathway of the innate immune system (G’al et al., 2009). Experimental evidence suggests that the *C1R* and/or *C1S* variants may cause extracellular presence of activated C1s without microbial triggers (Bally et al., 2019; Gröbner et al., 2019), which would lead to gingival hyperinflammation in response to mild biofilm accumulation, and subsequently rapidly progressing periodontal destruction. However, there are other pEDS clinical features unrelated to biofilm pathogens and apparently could not be explained by the above hypothesis and the detailed mechanisms remain largely unknown. To gain insights into altered gene expression patterns and dysregulated biological processes underlying molecular pathology of pEDS, we carried out transcriptome profiling by RNA sequencing of monocytes and gingival fibroblasts from two patients with pEDS compared with normal controls.

## Methods

### Study Approval

This study was conducted according to the Declaration of Helsinki for Human Rights and all procedures were reviewed and approved by the Ethics Committee of Nanjing Stomatological Hospital, Medical School of Nanjing University (2018NL-037). All participants provided written informed consent before their enrollment in the present study. Samples were de-identified before analysis.

### Participant Recruitment

Monocytes were obtained from two patients with pEDS from our previous study (Wu et al., 2018). Patients’ information was as follows: the proband IV-1 (male, 25 years old, referred as pEDS1 in this study) was found with both a missense mutation in *C1R* (c.265T > C) and a frameshift mutation in *COL3A1* (c.1322delG); the proband’s mother III-2 (female, 48 years old), referred as pEDS2 in the present study, only had the same mutation in *C1R*. Normal controls were periodontal healthy adults who showed no BOP, PD ≤ 3 mm, and no CAL, who underwent crown lengthening surgery for the restorative purpose in the Department of Periodontology, Nanjing Stomatological Hospital, Medical School of Nanjing University and exclusions included acute illness, pregnancy, and other systemic diseases.

Gingival tissues were obtained from two pEDS patients during their tooth extraction surgery, while control gingival tissues were obtained from three periodontal healthy adults during the crown lengthening surgery. All participants in the present study underwent clinical examination, and the detailed clinical data of all individuals involved in this study were recorded.

### Isolation and Culture of Monocytes Extracted from Human Blood Samples

Blood samples collected from two pEDS patients and three normal controls were used to extract peripheral blood mononuclear cells (PBMCs) using density gradient centrifugation. PBMCs of each group were added with RPMI1640 medium (Gibco) containing 2%FBS (Gibco) and incubated at 37°C with 5% CO2 for 2 h. Then the supernatant was harvested and stored for the later experiment of enzyme-linked immunosorbent assay (ELISA). The remained cells were isolated monocytes and washed twice by sterile PBS (Servicebio) gently. RPMI1640 medium supplemented with 10% FBS, 100 U/ml penicillin, and 100 μg/ml streptomycin (HyClone) for further culture for 12 h.

### Isolation and Culture of Human Gingival Fibroblasts

Gingival connective tissues were obtained from 2 pEDS patients during tooth extraction surgery and three normal controls during crown lengthening surgery. The collected gingival tissue was immersed in DMEM (Gibco) supplemented with 100 U/ml penicillin, and 100 μg/ml streptomycin (HyClone); and the tissue was cut into pieces, approximately 1 × 1 mm in size and placed in DMEM supplemented with 10% FBS, 100 U/ml penicillin, and 100 μg/ml streptomycin; tissue was then incubated at 37°C with 5% CO_2_, HGF at the $2^{nd}$ passage was harvested for future experiments.

### Gene Expression Profiling

Total RNA was isolated from monocytes and gingival fibroblasts by TRIzol reagent (Invitrogen) according to the manufacturer’s instructions. The RNA samples were quality assessed and the mRNA is enriched using magnetic beads with Oligo (dT). The mRNA was then broken into short fragments by adding fragmentation buffer, and one-stranded cDNA was synthesized using random hexamers as templates, followed by the addition of buffer, dNTPs, and DNA polymerase I and RNase H. The double-stranded cDNA was purified with AMPure XP beads and was end-repaired, added with polyA tails and adapters sequences. After size selection, PCR amplification, and purification, the library was finally obtained. After the quality assessment of the library, RNA sequencing was performed on an Illumina HiSeq X Ten instrument. The raw reads were cleaned by removing adapter sequences, trimming low-quality ends, and filtering low-quality reads (Phred quality <20) using TrimGalore (version 0.6.5). Transcriptome quantification of transcript expression was carried out by using the mapping-based mode of Salmon (version 1.5.2) with the pre-built version of the full-decoy salmon index provided by Salmon.

### RNA-Seq Data Analysis

Normalisation and differential expression between patients and normal controls were evaluated using DESeq2 (version 1.32.0) (Love et al., 2014), implemented in R (version 4.1.2). DESeq2 uses a count-based negative binomial model to detect differentially expressed genes (DEGs). DEGs were defined as genes with the adjusted *p*.value < 0.05 and the absolute value of fold change >1.5 $\left(|log_{2}FC\right|$ > 1.5).

Principal component analysis (PCA), dendrogram and hierarchical clustering heatmap were performed with the variance stabilization transformation values obtained by DESeq2 R package from the gene expression values: PCA was calculated for the whole dataset by using princomp function of R (Anders and Huber, 2010); The dendrogram was created by hclust function and ggplot2 package (version 3.3.5) (Wickham, 2016, p. 2); The hierarchical clustering heatmap was generated by ComplexHeatmap (version 2.10.0) package (Gu et al., 2016) using the top 100 DEGs.

Since there are only two pEDS samples and one of them was a rare case with both pEDS and vEDS, three-dimensional volcano plots were created with scatterplot3d (version 0.3–41) (Ligges & Mächler, 2003): the logarithm of fold change between pEDS1 and normal controls ( $\log_{2}FC_{pEDS1-normal}$ ) was represented on the x axis, the logarithm of fold change between pEDS2 and normal controls ( $\log_{2}FC_{pEDS2-normal}$ ) was represented on the y axis and the overall adjusted p.value was represented on the z axis. The filtering criteria included:1) The overall adjusted p.value <0.05; 2) | $\log_{2}FC_{pEDS1-normal}$ |>1.5 and 3) | $\log_{2}FC_{pEDS2-normal}$ |>1.5. The differential analysis in this step was performed with edgeR (version 3.36.0) (Robinson et al., 2010) and limma (version 3.50.0) (Ritchie et al., 2015).

For Over-Representation Analysis (ORA) and Gene Set Enrichment Analysis (GSEA), GO and KEGG enrichment analysis using detected DEGs and gene set enrichment analysis (GSEA) using ranked gene lists were performed with the software package clusterProfiler (Yu et al., 2012; Wu et al., 2021) (R-version 4.1.2, clusterProfiler_4.0.5) to identify enriched biological processes and molecular functions; The filtering standard for ORA and GSEA geneList is | $\log_{2}FC$ |>1.5. The query was carried out on all filtered DEGs by selecting a threshold of FDR-adjusted *p*-value less than 0.05. Similarly, the filtering adjusted *p*-value for GSEA should also be less than 0.05.

All the R packages we used in the analysis are listed in Supplementary Table S1.

### STRING Network Analysis

STRING networks can provide information on the molecular mechanism underlying clinical features. A STRING network of DEGs involved in periodontitis and genes involved in the classical complement activation pathway was constructed using the STRING protein query (Szklarczyk et al., 2021) (STRING Version 11.5) and Cytoscape software (Shannon et al., 2003; Doncheva et al., 2019) (Cytoscape Version 3.8.2). The lines represent interaction associations between nodes and line thickness indicates the strength of data support. Selected DEGs were mapped to STRING to identify the interactive relationships among those genes. A confidence score of 0.4 was set as the cut-off criterion, and the node size in periodontitis-related genes is mapped to the logFC values of gene expression value in monocytes. Additionally, an extended STRING network showing the interaction between classical complement pathway and all DEGs in this study was also created, which could be found in Supplementary Figure S2A. We also identified the top 10 hub genes and sub-networks by using a Cytoscape plugin cytoHubba (Supplementary Figure S2B) (Chin et al., 2014).

### Quantitative Reverse Transcription PCR for Validation

Relative expression levels of a series of selected DEGs (*MMP9*, *VEGFA*, *IL10*, *IL1A*, *IL1B*, *IL2RA,* and *IL6*) identified by RNA sequencing were confirmed by RT-qPCR using different RNA extractions obtained from monocytes cultures of corresponding pEDS and normal controls. 3 µg of total RNA were reverse-transcribed with random primers by standard procedure. RT-qPCR was performed with SYBR Green qPCR Master Mix (Life Technologies), 10 ng of Cdna, and with 10 μ M of each primer set. The experiment was performed using the ABI PRISM 7500 Real-Time PCR System by standard thermal cycling conditions: Preincubation for 30 s at 95°C, followed by 40 cycles of 95°C for 10 s and 60°C for 30 s. GAPDH and CYC1 reference genes were amplified for normalization of Cdna loading. All specific primers used in the present study were summarized in Supplementary Table S2.

### Enzyme-Linked Immunosorbent Assay

Enzyme-linked Immunosorbent Assay (ELISA) based quantification of IL-1β (Neobioscience Technology) concentration was measured using concentrated supernatants that we collected in monocytes isolation steps, following the supplier’s instructions.

### Statistics

For RT-qPCR, relative mRNA expression levels were normalized to the geometric mean of these reference genes and analyzed using the $2^{-△△Ct}$ method. Results were expressed as the mean value of relative quantification [mean (SD)]. Statistical significance between groups was determined using unpaired Student’s t-test (ns = not significant, * = *p* < 0.05, **p < 0.01, ***p < 0.001, ****p < 0.0001) with the R package ggpubr (Kassambara, 2020) (R-version 4.1.2, ggpubr_0.4.0). For ELISA, statistical significance between groups was determined with the R package ggpubr (Kassambara, 2020) (R-version 4.1.2, ggpubr_0.4.0). Results were tested for normality with Shapiro-Wilk’s test. Since not all sample groups passed the normality test, we applied the unpaired, non-parametric Wilcoxon test (ns = not significant, * = *p* < 0.05, **p < 0.01, ***p < 0.001, ****p < 0.0001).

## Results

### Participant Enrollment

Two pEDS patients and three periodontal healthy adults (two males at 25 and 28 years old as normal1 and normal2 respectively; one female at 25 years old as normal3) were recruited in this study. The detailed clinical data of all individuals involved in this study were summarized in Table 1, oral photographs of all individuals were available in Supplementary Figure S1. As described in our previous work (Wu et al., 2018), it is worth noting that pEDS1 also carried a frameshift variant in *COL3A1* and thus might be affected by vascular EDS (vEDS).

**TABLE 1:** The clinical findings of all individuals including age, gender, gingiva specimen sampling position, and periodontal examination results.

ID	Age (y)	Sex	Sampling	BOP	PD (mm)			CAL (mm)		
			Site							
pEDS1	25	Male	13	+	15	11	10	15	13	13
					13			13		
					5	6	14	5	6	14
pEDS2	48	Female	13	+	7	3	5	13	6	5
					13			13		
					6	2	4	9	4	5
Normal1	25	Male	11	-	3	3	2	0	0	0
					11			11		
					2	1	2	0	0	0
Normal2	28	Male	21	-	2	3	1	0	0	0
					21			21		
					2	2	2	0	0	0
Normal3	25	Female	11	-	2	2	2	0	0	
					11			11		
					2	1	2	0	0	0

### Quality Assessment of Transcriptome Profiling

**FIGURE 1:**
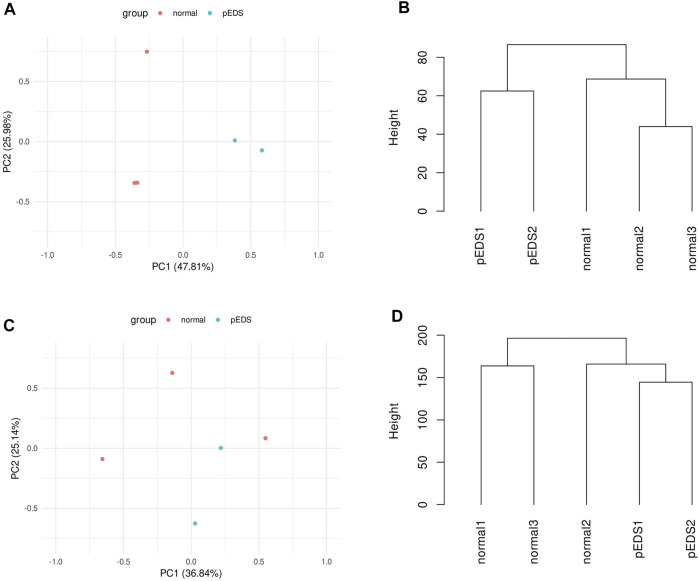
The correlation between pEDS and control group was evaluated by quality assessment methods including principal component analysis (PCA) and dendrogram. **(A)** PCA shows 73.8% of the variability within the monocyte samples is accounted by two principal components. Note the clustering of pEDS (green) vs. normal controls (red); **(B)** For monocytes, dendrogram shows a clear clustering of the controls and the pEDS subjects based on gene expression values; **(C,D)** As for HGF samples, the first two principal components can not separate pEDS (green) from normal controls (red); and samples from same group do not cluster. Dendrogram, consistent with PCA result, does not show a significant clustering pattern.

The correlation between pEDS and control group was evaluated by quality assessment methods including principal component analysis (PCA) and dendrogram. For the monocytes, PCA shows two principal components that could account for 73.8% of the variability between the samples (Figure 1A). The dendrogram of hierarchical clustering (Figure 1B) show a clear clustering of the controls and the pEDS patients based on gene expression values. However, for the HGF, no significant pattern can be found in the clustering of gene expression values between different samples (Figures 1C,D). The normalized count matrix files of monocyte and fibroblast can be found in Supplementary Tables S3, S4.

### Differential Expression Analysis

**FIGURE 2:**
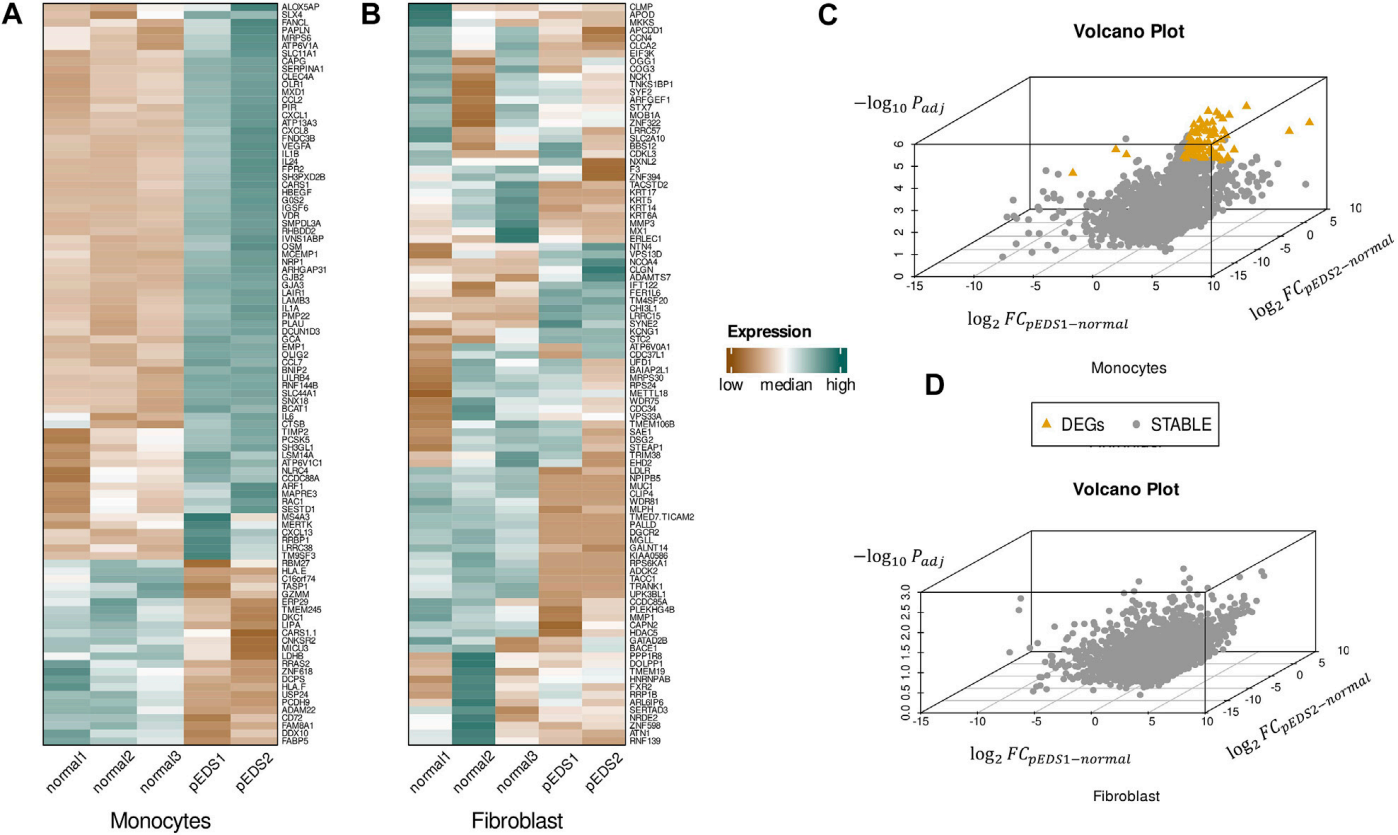
Three dimensional volcano plot and hierarchical heatmap of the DEGs found within the monocyte and gingival fibroblast samples. **(A,B)** Hierarchical heatmap of the top 100 differential expressed genes within the monocyte and gingival fibroblast samples. Red denotes increased expression, and green denotes decreased expression. A significant pattern can only be observed in monocytes but not in gingival fibroblasts. **(C,D)** Three-dimensional volcano plots for monocytes and fibroblasts: $\log_{2}FC_{pEDS1-normal}$ was represented on the x axis, $\log_{2}FC_{pEDS2-normal}$ was represented on the y axis and the overall adjusted *p*.value was represented on the z axis. The filtering criteria included:1) The overall adjusted *p*.value <0.05; 2) | $\log_{2}FC_{pEDS1-normal}$ |>1.5 and 3) | $\log_{2}FC_{pEDS2-normal}$ |>1.5. Orange triangle denotes DEGs, gray point denotes not differentially expressed genes. DEGs were only found in monocytes.

Differentially expressed genes (DEGs) were identified by DESeq2 R package according to the filtering criteria. Approximately 3% of the detected transcriptome showed differential expression in monocytes and there were 338 DEGs in pEDS patient-derived monocytes, of which 246 genes were up-regulated and 92 genes were down-regulated. The complete list of DEGs could be found in Supplementary Table S5. Hierarchical heatmaps of the top 100 differential expressed genes within the monocyte and gingival fibroblast samples were shown in Figures 2A,B, which implied that a significant pattern can only be observed in monocytes but not in gingival fibroblasts. In addition, it should be noted that C1R and C1S were not differentially expressed in both monocytes and gingival fibroblasts from pEDS group.

Since one of two pEDS patients was a rare case with both pEDS and vEDS, three-dimensional volcano plots were created to present the DEGs (Figures 2C,D): A total of 92 DEGs were identified in monocytes but no DEGs could be found in HGF. It should also be noted that 45 DEGs could be identified between sample pEDS1 and pEDS2 but no significant result was identified in the subsequent ORA and GSEA analysis (data not shown).

### Over-Representation Analysis and Gene Set Enrichment Analysis of Differentially Expressed Genes in Monocytes.

**FIGURE 3:**
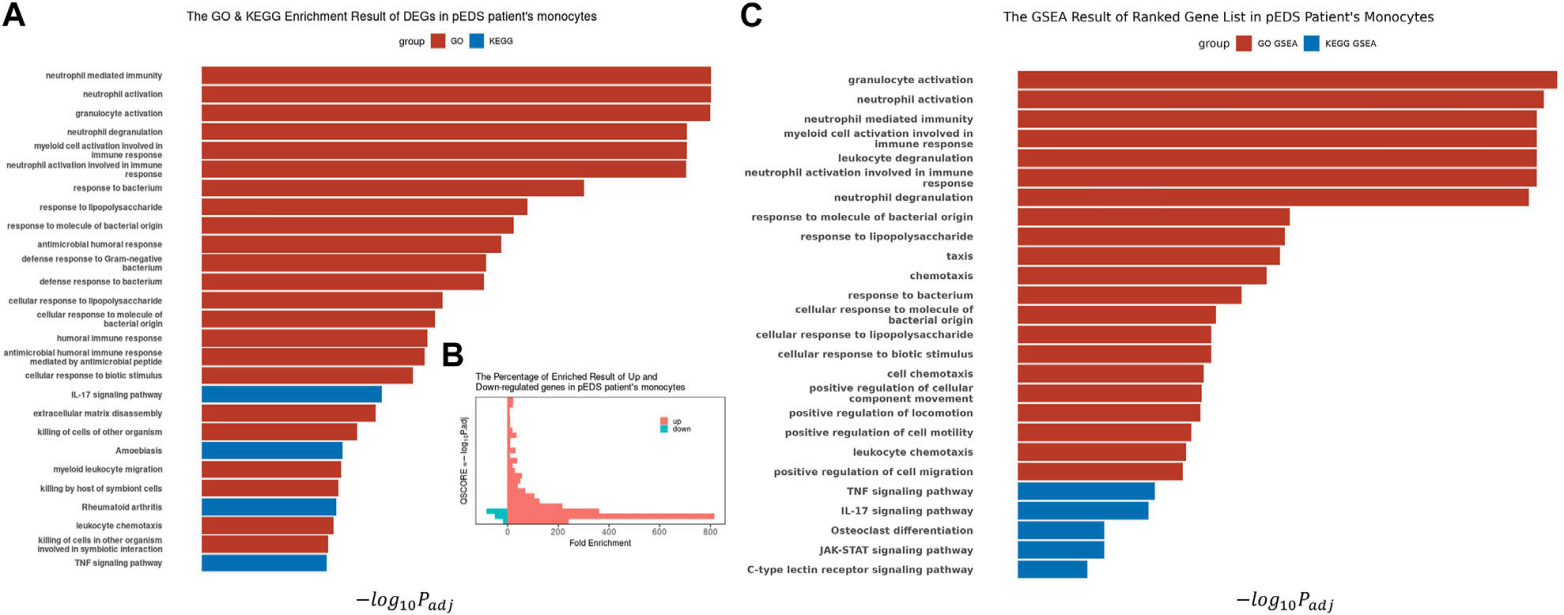
The Biological Processes GO Enrichment Result and GSEA result of DEGs in pEDS patients’ monocytes. **(A)** Biological processes related to inflammation response and immune response are significantly enriched; **(B)** Most of the significant results are contributed by upregulated genes, which also have higher Fold Enrichment (defined as GeneRatio/BgRatio in clusterProfiler) compared to downregulated genes; **(C)** The GSEA analysis result also showed similar enriched result in pathways related to immunity, inflammation, IL-17 pathway and TNF-α pathway.

With the identified 338 DEGs, GO and KEGG enrichment analysis, as well as GSEA, were performed using the software package clusterProfiler (Version 4.0.5) (Yu et al., 2012; Wu et al., 2021). Most of the significant results are enriched in up-regulated genes (Figure 3B), especially in the pathway of biological processes, which are mainly related to inflammation response and immune response (Figure 3A). This finding is consistent with GSEA results (shown in Figure 3C), which are also highly enriched in pathways with positive enrichment scores (NES). Combined with differential expression analysis, it could be identified that some DEGs, i. e, RETN, DEFA1, ANXA3, LTF, and LCN2, are specifically involved in neutrophil, myeloid cell activation, and mediated immunity events. The complete list of GO-GSEA results were summarized in Supplementary Table S6. For the KEGG enrichment analysis, the most significant results were focused on the IL-17 signaling pathway and TNF signaling pathway. The complete enriched results of GO and KEGG analysis are summarized in Supplementary Table S7.

### The Disease Ontology Enrichment Analysis of DEGs in Monocytes and Validation by Reverse Transcription-qPCR and Enzyme-Linked Immunosorbent Assay

**FIGURE 4:**
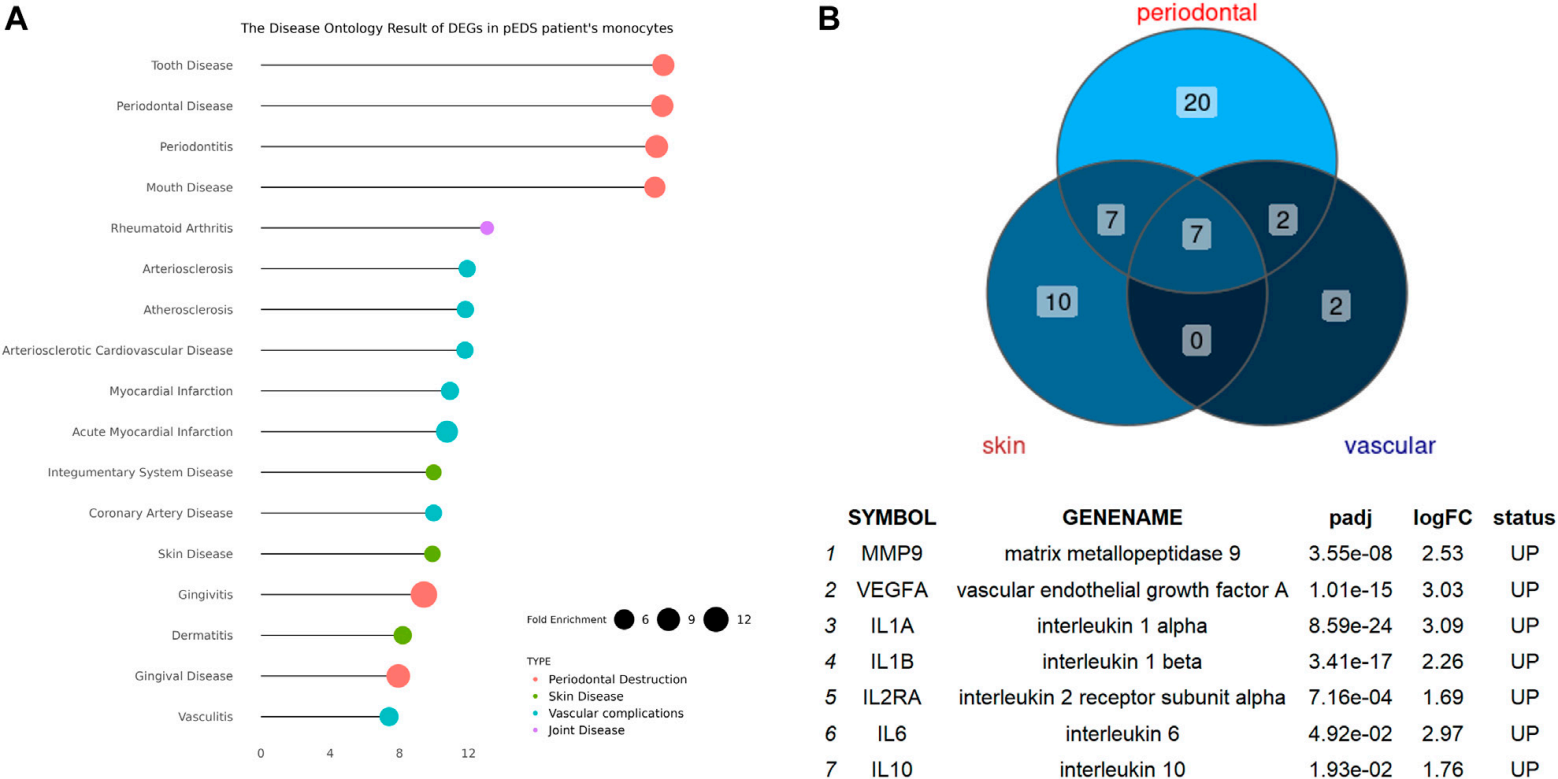
The Disease Ontology Enrichment Analysis Result of DEGs in pEDS patients’ monocytes and the overlapped enriched genes between DO categories of periodontal destruction, vascular complication, and skin disease in pEDS patients’ monocytes. **(A)** The DO results can be divided into four categories which are separately related to different clinical features of pEDS: periodontal destruction, skin fragility, vascular complication, and joint hypermobility (Colors represent different clinical features of pEDS; Horizontal axis represent Fold Enrichment of each DO category, which is defined as GeneRatio/BgRatio in clusterProfiler); **(B)** seven genes including *MMP9, VEGFA, IL10, IL1A, IL1B, IL2RA*, and *IL6* have been found as the overlapped genes in three different DO categories and all of them were significantly upregulated in monocytes of pEDS.

**FIGURE 5:**
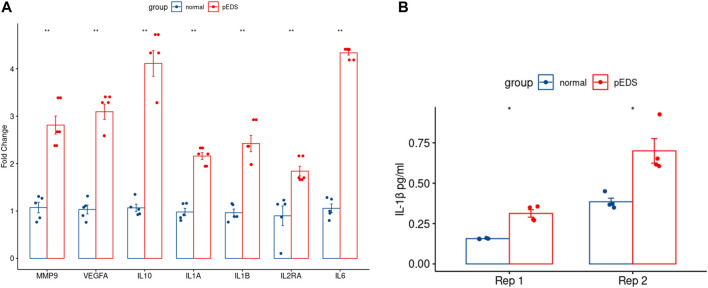
RT-qPCR of the overlapped genes and the ELISA result of IL-1β expression in pEDS patients’ monocytes were performed to validate the RNA sequencing results of pEDS patients. **(A)** In RT-qPCR analysis, gene expression levels were measured in four independent replicates per subject, and t-tests were performed (ns = not significant, *p < 0.05, **p < 0.01, ***p < 0.001, ****p < 0.0001). All seven genes including *MMP9, VEGFA, IL10, IL1A, IL1B, IL2RA*, and *IL6* are significantly expressed in pEDS group (*p* < 0.01); **(B)** For ELISA experiment, data are expressed as mean (SD). (ns = not significant, *p < 0.05, **p < 0.01, ***p < 0.001, ****p < 0.0001). IL-1β concentration was also significantly higher in the monocytes of pEDS patients (*p* < 0.05).

The detailed mechanism behind complement pathway disruption and related pEDS clinical manifestations is not yet explained. Disease Ontology (DO) was developed to create a consistent description of gene products with disease perspectives, and accurate disease descriptions can discover new relationships between genes and disease (Schriml et al., 2012). Thus, we performed DO analysis with upregulated DEGs to help us to better understand the relationship between the mutant variants and pEDS. As shown in Figure 4A, the DO enriched results can be divided into several categories, which is separately related to different aspects of clinical features of pEDS: periodontal destruction, skin fragility, vascular complication, and joint hypermobility [Colors represent different clinical features of pEDS; Horizontal axis represent Fold Enrichment of each DO category, which is defined as GeneRatio/BgRatio in clusterProfiler (Yu et al., 2015)]. Besides, to identify genes related to the pathogenesis of pEDS, the overlapping between DO categories of periodontal destruction, vascular complications, and skin disease were selected (Figure 4B). The complete list of DO terms of upregulated DEGs is shown in Supplementary Table S8. The selected DEGs including *MMP9*, *VEGFA*, *IL10*, *IL1A*, *IL1B*, *IL2RA,* and *IL6* were identified and validated by RT-qPCR. All 7 genes are significantly expressed in the pEDS group compared to controls (Figure 5A). MMP9, IL10, IL1A, IL1B, IL6 were also measured as the top 10 hub genes (Supplementary Figure S2B) by cytoHubba (Chin et al., 2014), confirming their important roles in pathomechanism of pEDS. ELISA was also performed to measure the IL-1 β concentration of supernatants collected in monocytes isolation steps (Figure 5B). Similar to the qPCR result, the IL-1β concentration was also significantly higher in the monocytes of pEDS patients.

### STRING Network

**FIGURE 6:**
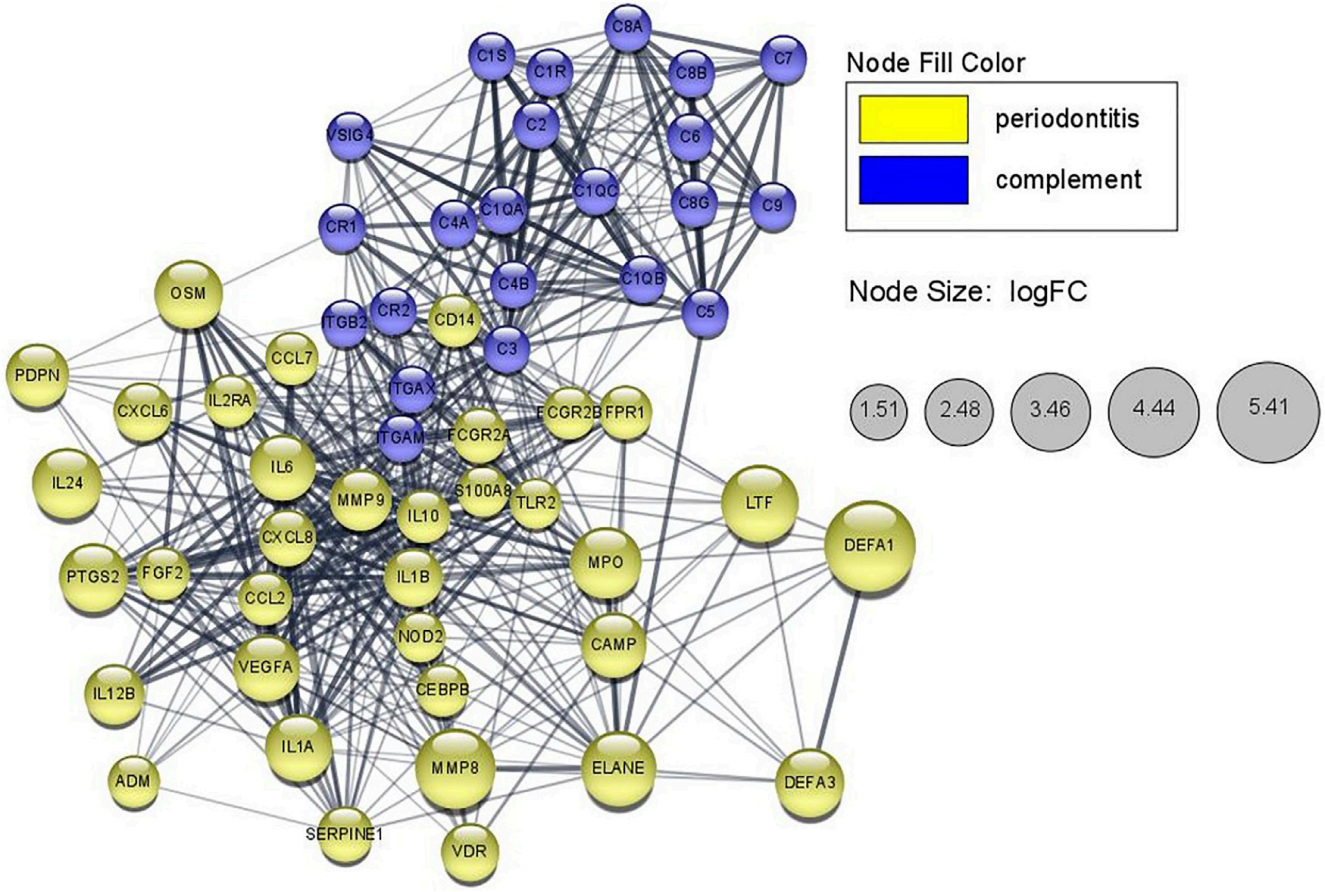
STRING network showing periodontitis-related proteins that possibly interacted with the classical complement pathway in monocytes of pEDS patients. The lines represent interaction associations between nodes and line thickness indicates the strength of data support. The node size in periodontitis-related genes is mapped to the logFC values of gene expression value in monocytes of pEDS patients.

As shown in Figure 6, the STRING network shows the relationship between genes involved in the classical complement activation pathway and the DEGs related to periodontitis. The overlapped genes from DO analysis including *MMP9*, *VEGFA*, *IL10*, *IL1A*, *IL1B*, *IL2RA*, *IL6* are also in the STRING networks and showed closer interaction among the nodes. The pathogenic mutant variant, *C1R/C1S* were located the upstream of STRING network and not directly connected to the periodontitis-related genes. Additionally, in the extended STRING network consisted of classical complement pathway and DEGs in this study (Supplementary Figure S2A), the nodes of C1R/C1S only connected to other complement components but not any DEG, which implied that the extracellularly active C1s protein might have other potential targets that has yet been found (Bally et al., 2019).

## Discussion

Transcriptomics have been utilized to disclose the key alterations of biological processes triggering human diseases, thus offering novel instruments useful not only for the comprehension of their underlying mechanisms but also for their molecular diagnosis and clinical therapy (Casamassimi et al., 2017). Previous studies (Chiarelli et al., 2016, 2018, 2019; Lim et al., 2019) performed transcriptome analysis on other EDS subtypes, which succeeded in revealing some of the mechanisms underlying these rare diseases. Up until now, the pathophysiology of pEDS is not well understood, and the current study described the first molecular evidence of significant gene expression changes in pEDS cells (monocytes and gingival fibroblasts) that could provide insights into the pathogenesis of the disease. Although the sample size is small and the findings will therefore need to be confirmed in other patients, our results provide a step forward towards understanding of the complex pathogenetic basis of pEDS. Multiple DEGs were identified only in monocytes but not in gingival fibroblasts. Approximately 3% of the detected transcriptome showing differential expression in monocytes and a total of 338 DEGs were identified in monocytes compared to normal control. ORA and GSEA analysis observed changes in neutrophil-mediated immunity, response to bacterium, humoral immune response, IL-17 pathway, and TNF-α pathway, of which are related to immune response and inflammatory response; DO analysis also identified potential target genes including *MMP9*, *VEGFA*, *IL10*, *IL1A*, *IL1B*, *IL2RA*, and *IL6*, which may be related to periodontal destruction, vascular complication, and skin disease of pEDS; In addition, STRING network analysis showed the relationship between genes involved in the classical complement activation pathway and the DEGs related to periodontitis. The overlapped genes from DO analysis are also in the STRING networks and some of them are considered as hub genes which highly interacted with other DEGs. This finding implied that these genes might play important roles in the pathophysiology of pEDS.

### IL-17 Pathway and Periodontal Destruction

In ORA and GSEA, genes related to the IL-17 pathway were enriched in pEDS monocytes. Previous studies has reported that, IL-17, IL-17–producing lymphocytes and innate immune cells are potentially important players in the pathogenesis of periodontitis (Hajishengallis, 2014). Besides, elevated expression level of IL-17 has been closely related to periodontitis (Ohyama et al., 2009; Darveau, 2010). Overall, IL-17 is a double-edged sword when it comes to inflammatory illnesses like periodontitis (Zenobia & Hajishengallis, 2015).

On the one hand, IL-17 has been demonstrated to protect against extracellular pathogens (Khader et al., 2009; Hernandez-Santos and Gaffen, 2012) and can trigger the generation of antimicrobial peptides (Liang et al., 2010), which are assumed to be protective in periodontitis (Diamond et al., 2008; Gorr, 2009). On the other hand, through upregulating matrix metalloproteases and RANKL, IL-17 can also facilitate connective tissue degradation and bone resorption (Lubberts, 2008). IL-17 could also facilitate neutrophil recruitment and increase inflammation by downregulating the endogenous anti-inflammatory molecule Del-1 (Eskan et al., 2012).

IL17 can also be involved in periodontitis development through the complement pathway. Complement and IL-17 are both involved in the regulation of neutrophil recruitment, which is an important mechanism in maintaining periodontal homeostasis (Eskan et al., 2012; Hajishengallis and Hajishengallis, 2014). It is also reported that single nucleotide polymorphisms in the components C5 and IL-17 have been linked to a higher risk of periodontal disease (Chai et al., 2010a; Corrêa et al., 2012; Kadkhodazadeh et al., 2013), implying that both molecules may play a role in the illness’s development. Although complement has a complex effect on IL-17 expression, it has been observed that complement, in collaboration with Toll-like receptors, can increase IL-17 synthesis in murine periodontal tissue (Abe et al., 2012). Mice lacking either C5Ar or Toll-like receptor-2, on the other hand, are protected from experimental periodontitis (Hajishengallis et al., 2011; Liang et al., 2011). In pEDS, the extracellularly activated C1s can activate the classical complement cascade and cleave more C4 and C2, which are components of the C3 convertase complex in the classical pathway (Bally et al., 2019). This disruption might ultimately induce the production of C5a (Ricklin and Lambris, 2013). With the support of Toll-like receptor-2 (TLR2 is also one of the upregulated DEGs and top 10 hub genes found in monocytes), abundant C5a may activate C5aR and upregulate IL-17, IL-1, IL-6, and TNF (Abe et al., 2012). Ultimately, IL-17 could facilitate neutrophil recruitment and result in periodontal destruction in synergy of other cytokines (Abe et al., 2012).

### Selected DEGs and Periodontal Destruction

Apart from IL-17, other upregulated genes we identified in DO analysis also can be potential players in the periodontal destruction of pEDS. For upregulated MMP9, it has been suggested that matrix metalloproteinases (MMPs) are key proteases involved in destructive periodontal diseases (Buduneli and Kinane, 2011; Sorsa et al., 2011). The most prevalent MMPs in periodontal tissues are MMP-8 and MMP-9, which reflect the severity, development, and treatment response of periodontal disease (Mäntylä et al., 2006). MMP-9 has been linked to periodontal soft tissue degradation and has been found to work with MMP-13 in alveolar bone resorption and periodontal tissue distruction (Hernández et al., 2006; Hernández et al., 2011).

IL-1α, IL-1β and IL-6 are proinflammatory cytokines that are thought to play a role in periodontitis development. Nonsurgical periodontal therapy has been shown to result in a statistically significant reduction in overall levels of these cytokines in gingival crevicular fluid (GCF) (Reis et al., 2014). Nonetheless, IL-6 and IL-10 polymorphisms are found to be potential risk factors for periodontitis (Scapoli et al., 2012). In addition, Afacan et al. reported that the concentrations of VEGF and TNF-α in GCF were significantly higher in the periodontitis group than in the gingivitis and healthy groups (Afacan et al., 2018). Total amounts of VEGF and TNF-α in GCF were positively correlated with the site-specific clinical periodontal parameters and with each other. Increased GCF VEGF and TNF-α levels in both chronic and aggressive forms of periodontitis might suggest the role of the TNF-α/VEGF pathway in the pathogenesis of periodontal diseases (Afacan et al., 2018). In conclusion, the up-regulation of these selected DEGs is strongly connected to the pathogenesis of periodontitis and has the potential to be the possible pharmacological target.

### Classical Complement Pathway and Periodontitis

The gain-of-function *C1R* and/or *C1S* variants are now recognized to be the essential element in the pathogenesis of pEDS. The extracellular presence of activated C1s can activate the conventional complement cascade without the presence of other signals (Gröbner et al., 2019). *In vitro* studies suggests that the production or release of active C1r serine protease may promote gingival hyperinflammation in response to mild biofilm, leading to severe periodontal damage (Bally et al., 2019; Gröbner et al., 2019).

The potential role of complement in human periodontitis was first recognized in the 1970s and 1980s when researchers looked into the GCF under different periodontal statuses (Attstroum et al., 1975; Courts et al., 1977; Schenkein and Genco, 1977). GCF samples from periodontitis patients were found to have complement-dependent hemolytic activity, indicating that GCF contains a functional complement system (Courts et al., 1977; Boackle, 1991). Recent studies also suggest an association between complement and periodontitis. A rare case of aggressive periodontitis with gingival angioedema was linked to deficiency of the C1INH (Roberts et al., 2003). People with periodontitis had a significantly higher occurrence of a single nucleotide polymorphism affecting C5 (rs17611), compared to healthy controls (Chai et al., 2010b). Another study identified C3 as one of the top 21 most promising candidate genes involved in periodontal disease using microarray experiments (Zhan et al., 2014). As for C5, it was reported that C5a can induce the activation of C5Ar in a murine periodontal disease and cause significant bone loss with the help of cytokines like IL-17, IL-1β, IL-6, and TNF (Abe et al., 2012). Conversely, C4 might have a protective effect against periodontitis since partial C4 gene deficiencies are significantly more common in periodontitis patients than in healthy individuals (Seppänen et al., 2007).

In this study, STRING network analysis showed a close relationship between genes involved in the classical complement pathway and DEGs in this study. C3, as previously reported, was located as the central element in the complement pathway (Ricklin and Lambris, 2013) and also showed closer links to other nodes in the STRING network. Meanwhile, *C1R* and *C1S* were upstream of the classical complement pathway and not directly connected to DEGs according to the evidences provided by STRING. Several DEGs such as MMP9, VEGFA, IL10, IL1A, IL1B, and IL6 that validated by RT-qPCR were also included in the STRING network and showed close connections to the complement pathway, which implied that they may play as important regulators between *C1R*/*C1S* mutation and downstream periodontal phenotypes. However, further research is necessary to confirm the relationship between genes involved in the classical complement pathway and our selected DEGs.

### Limitations

Firstly, we acknowledged that our study has a small sample size (due to patient cohort size), which may affect the power of some observations. In addition, only two pEDS patients were enrolled and one of them was affcted by both pEDS and vEDS, which means our result need to be confirmed in other patients in further research. Besides, compared to previous transcriptomic profiling of other EDS subtypes (Chiarelli et al., 2016, 2018, 2019), we used gingival fibroblasts rather than skin fibroblasts. It is not certain that other ECM producing cells, i.e., vascular smooth muscle cells, and skin fibroblasts, would show the same results in terms of gene expression profile. Finally, in the present study, we only validated the RNA-sequencing result by RT-qPCR and ELISA. Further research must be designed and performed to validate the results we found in this study.

## Conclusion

In conclusion, our approach illustrates global Mrna profiling changes of several genes and related biological processes that could offer novel insights into the pEDS pathophysiology. Approximately 3% of the detected transcriptome showed differential expression and were identified only in monocytes but not in gingival fibroblasts, multiple DEGs were enriched in neutrophil-mediated immunity, response to bacterium, humoral immune response, IL-17 pathway, and TNF- α pathway. Potential target genes including *MMP9*, *VEGFA*, *IL10*, *IL1A*, *IL1B*, *IL2RA*, and *IL6* were significantly upregulated in monocytes which related to periodontal destruction, vascular complication, and skin disease. However, additional functional work is required to verify whether these up-regulated factors are essential in the pEDS mechanism.

## Data Availability

The datasets presented in this study can be found in online repositories. The names of the repository/repositories and accession number(s) can be found below: https://www.ncbi.nlm.nih.gov/geo/query/acc.cgi?acc=GSE190786.

## Supplementary Material

Supplementary Figure S1 Oral photographs of all individuals enrolled in this study.

Supplementary Figure S2 The extended STRING network as well as the top 10 hub genes calculated by cytoHubba. A: The STRING network constructed by the classical complement pathway and all DEGs. C1R/C1S nodes only showed direct connections to other complement component. B: Top 10 hub genes included *CCL2, CXCL1 ,CXCL2, CXCL8, IL1A, IL1B, IL6, IL10, MMP9* and *TLR2*. the color from yellow to red denotes the degree of hub genes interacted with other DEGs.

Supplementary Table S1 All the R packages and software used for RNA sequencing data analysis in this study.

Supplementary Table S2 Specific gene primers used for RT-qPCR in the study.

Supplementary Table S3 The normalized read counts of monocytes.

Supplementary Table S4 The normalized read counts of fibroblasts.

Supplementary Table S5 List of DEGs that are up-regulated and down-regulated in pEDS patients’ monocytes over controls.

Supplementary Table S6 The complete list of GO-GSEA results in pEDS patients’ monocytes over controls.

Supplementary Table S7 The complete list of enriched GO and KEGG terms of DEGs in pEDS patients’ monocytes over controls.

Supplementary Table S8 The complete list of enriched DO terms of upregulated DEGs in pEDS patients’ monocytes over controls.
