# Supplementary material for: Transcriptome Analysis of Monocytes and Fibroblasts Provides Insights Into the Molecular Features of Periodontal Ehlers-Danlos Syndrome
Source: Front Genet. 2022 Apr 28;13:834928. doi: 10.3389/fgene.2022.834928 (PMC9095904; doi:10.3389/fgene.2022.834928)
Supplement: Supplementary file 4 [file Table1.DOCX]

We used R (Version 4.1.2; R Core Team, 2021) and the R-packages *AnnotationDbi* (Version 1.54.1; Hervé Pagès, Carlson, Falcon, & Li, 2021), *Biobase* (Version 2.52.0; Huber et al., 2015a), *BiocGenerics* (Version 0.38.0; Huber et al., 2015b), *circlize* (Version 0.4.13; Gu, Gu, Eils, Schlesner, & Brors, 2014), *citr* (Version 0.3.2; Aust, 2019), *clusterProfiler* (Version 4.0.5; T. Wu et al., 2021; Yu et al., 2012), *cowplot* (Version 1.1.1; Wilke, 2020), *DESeq2* (Version 1.32.0; Love et al., 2014), *DOSE* (Version 3.18.3; Yu, Wang, Yan, & He, 2015), *dplyr* (Version 1.0.7; Wickham, François, Henry, & Müller, 2021), *edgeR* (McCarthy, Chen, & Smyth, 2012; Version 3.34.1; Robinson, McCarthy, & Smyth, 2010), *EnhancedVolcano* (Version 1.10.0; Blighe, Rana, & Lewis, 2021), *enrichplot* (Version 1.12.3; Yu, 2021), *forcats* (Version 0.5.1; Wickham, 2021a), *GenomeInfoDb* (Version 1.28.4; Arora, Morgan, Carlson, & Pagès, 2021), *GenomicFeatures* (Version 1.44.2; Lawrence et al., 2013a), *GenomicRanges* (Version 1.44.0; Lawrence et al., 2013b), *ggdendro* (Version 0.1.22; de Vries & Ripley, 2020), *ggforce* (Version 0.3.3; Pedersen, 2021), *ggfortify* (Version 0.4.13; Tang, Horikoshi, & Li, 2016), *ggplot2* (Version 3.3.5; Wickham, 2016), *ggpubr* (Version 0.4.0; Kassambara, 2020), *ggrepel* (Version 0.9.1; Slowikowski, 2021), *ggsci* (Version 2.9; Xiao, 2018), *ggVennDiagram* (Version 1.2.0; Gao, 2021), *gridExtra* (Version 2.3; Auguie, 2017), *IRanges* (Version 2.26.0; Lawrence et al., 2013c), *kableExtra* (Version 1.3.4; Zhu, 2021), *koRpus* (Michalke, 2020a, Version 0.13.8; 2021), *koRpus.lang.en* (Version 0.1.4; Michalke, 2020a), *limma* (Version 3.48.3; Ritchie et al., 2015), *magrittr* (Version 2.0.1; Bache & Wickham, 2020), *MatrixGenerics* (Version 1.4.3; Ahlmann-Eltze, Hickey, & Pagès, 2021), *matrixStats* (Version 0.61.0; Bengtsson, 2021), *msigdbr* (Version 7.4.1; Dolgalev, 2021), *org.Hs.eg.db* (Version 3.13.0; Carlson, 2021), *pacman* (Version 0.5.1; Rinker & Kurkiewicz, 2018), *papaja* (Version 0.1.0.9997; Aust & Barth, 2020), *patchwork* (Version 1.1.1; Pedersen, 2020), *pheatmap* (Version 1.0.12; Kolde, 2019), *purrr* (Version 0.3.4; Henry & Wickham, 2020), *RColorBrewer* (Version 1.1.2; Neuwirth, 2014), *readr* (Version 2.1.1; Wickham & Hester, 2021), *readxl* (Version 1.3.1; Wickham & Bryan, 2019), *reshape2* (Version 1.4.4; Wickham, 2007), *rmdfiltr* (Version 0.1.3; Aust, 2021), *S4Vectors* (Version 0.30.2; H. Pagès, Lawrence, & Aboyoun, 2021), *shiny* (Version 1.7.1.9002; Chang et al., 2021), *stringr* (Version 1.4.0; Wickham, 2019), *SummarizedExperiment* (Version 1.22.0; Morgan, Obenchain, Hester, & Pagès, 2021), *sylly* (Version 0.1.6; Michalke, 2020b), *tibble* (Version 3.1.6; Müller & Wickham, 2021), *tidyr* (Version 1.1.4; Wickham, 2021b), *tidyverse* (Version 1.3.1; Wickham et al., 2019), *tinylabels* (Version 0.2.1; Barth, 2021), and *tximport* (Version 1.20.0; Soneson, Love, & Robinson, 2015) for all our analyses.
