# Supplementary figures and images for: Transcriptome Analysis of Monocytes and Fibroblasts Provides Insights Into the Molecular Features of Periodontal Ehlers-Danlos Syndrome
Source: Front Genet. 2022 Apr 28;13:834928. doi: 10.3389/fgene.2022.834928 (PMC9095904; doi:10.3389/fgene.2022.834928)

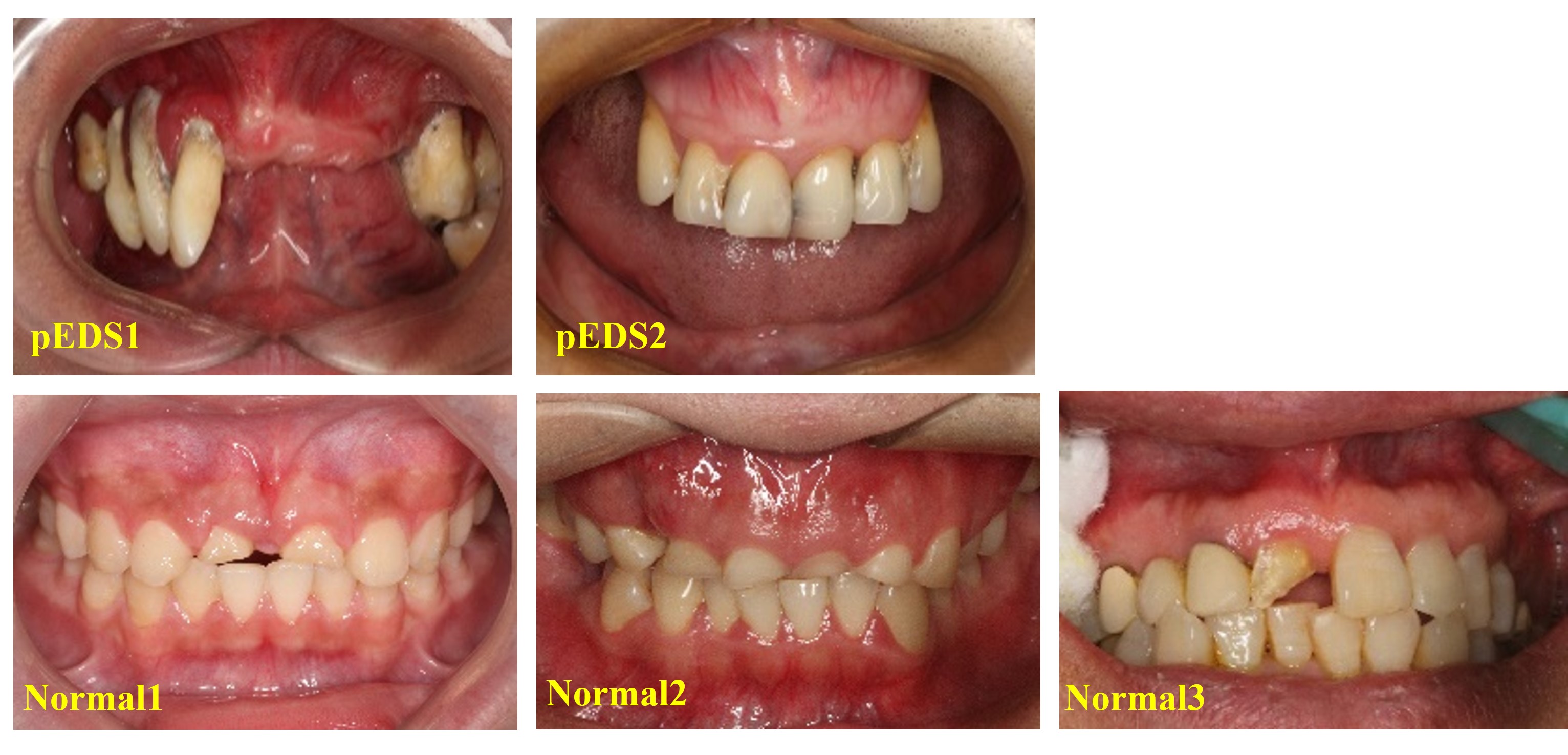

Supplement: Supplementary file 5 [file Image1.JPEG]

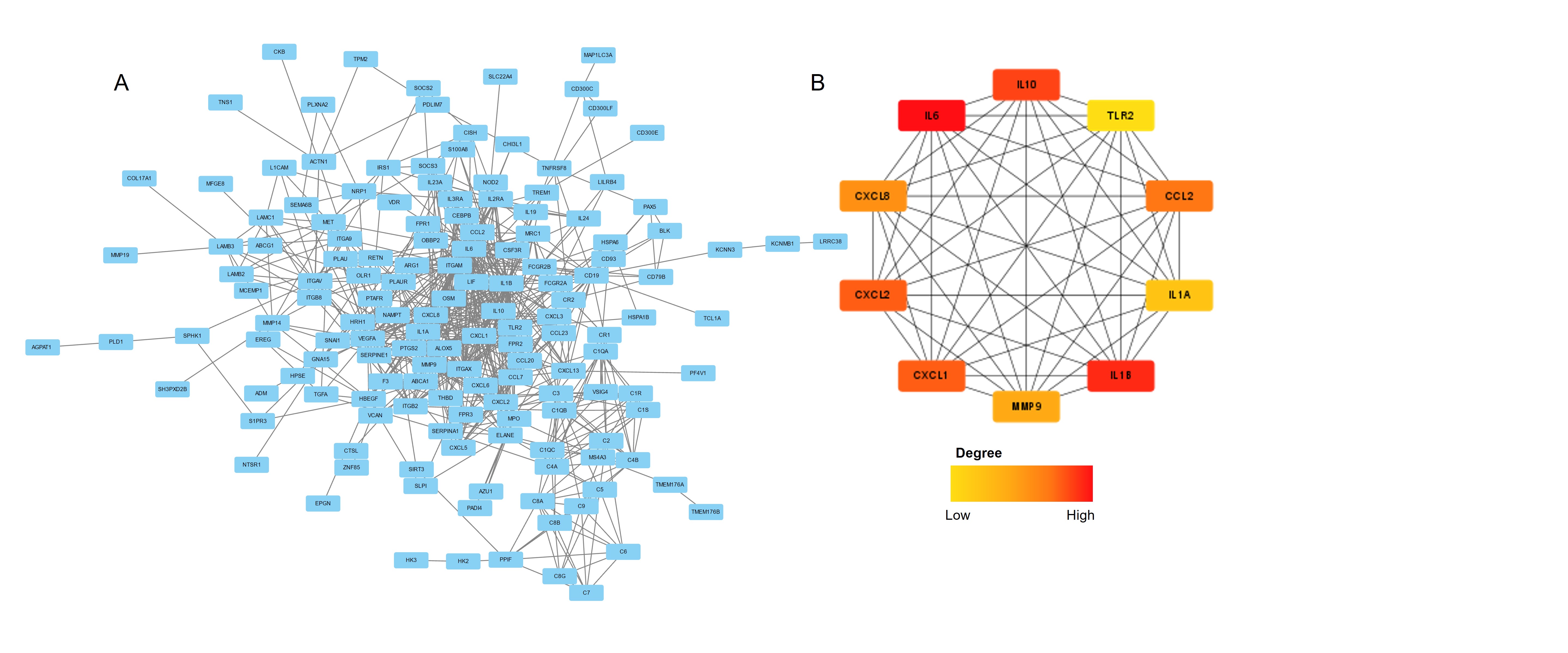

Supplement: Supplementary file 6 [file Image2.JPEG]
