# Supplementary material for: Transcriptome Analysis of Monocytes and Fibroblasts Provides Insights Into the Molecular Features of Periodontal Ehlers-Danlos Syndrome
Source: Front Genet. 2022 Apr 28;13:834928. doi: 10.3389/fgene.2022.834928 (PMC9095904; doi:10.3389/fgene.2022.834928)
Supplement: Supplementary file 7 [file Table2.DOCX]

| **Gene** | | **NCBI RefSeq** | **Primer forward** | **Primer reverse** |
| --- | --- | --- | --- | --- |
| **GAPDH** | NM_002046.7 | CCATGGGGAAGGTGAAGGTC | AGTGATGGCATGGACTGTGG |  |
| **CYC1** | NM_001916.5 | CTTCGCGGGGTAGTGTTGG | TAGCTCGCACGATGTAGCTG |  |
| **IL1A** | NM_000575.5 | AGATGCCTGAGATAACCCAAAACC | CCAAGCACACCCAGTAGTCT |  |
| **IL1B** | NM_000576.3 | ATGATGGCTTATTACAGTGGCAA | GTCGGAGATTCGTAGCTGGA |  |
| **IL2RA** | NM_000417.3 | CCCACACGCCACATTCAAAG | TTGTGTTCCGAGTGGCAGAG |  |
| **IL6** | NM_000600.5 | ACTCACCTCTTCAGAACGAATTG | CCATCTTTGGAAGGTTCAGGTTG |  |
| **IL10** | NM_000572.3 | TGCTCTTGCAAAACCAAACCA | GGGAGGTCAGGGAAAACAGC |  |
| **VEGFA** | NM_001171623.2 | TTGGAAACCAGCAGAAAGAG | CCAAAAGCAGGTCACTCACT |  |
| **MMP9** | NM_004994.3 | AAGGGTACAGCCTGTTCCTGGT | CTGGATGCCGTCTATGTCGTCT |  |
